# MRI AI Use Case: Synthetic CT Images for Fracture Evaluation

**DOI:** 10.5334/jbsr.2780

**Published:** 2022-06-22

**Authors:** Thiebault Saveyn, Lennart Jans, Frederiek Laloo

**Affiliations:** 1 UZ Gent, BE

**Keywords:** MRI, synthetic CT, artificial intelligence, bone imaging, fracture

## Abstract

**Teaching Point:** Synthetic CT images can improve evaluation of bony lesions on MRI and show potential for fracture evaluation, but validation is needed.

## Case History

A 20-year-old woman was referred to the emergency department after a fall from a height of 4 meters. She had severe pelvic pain and could not bear weight on the left leg. X-rays showed no fracture.

**Figure 1:**
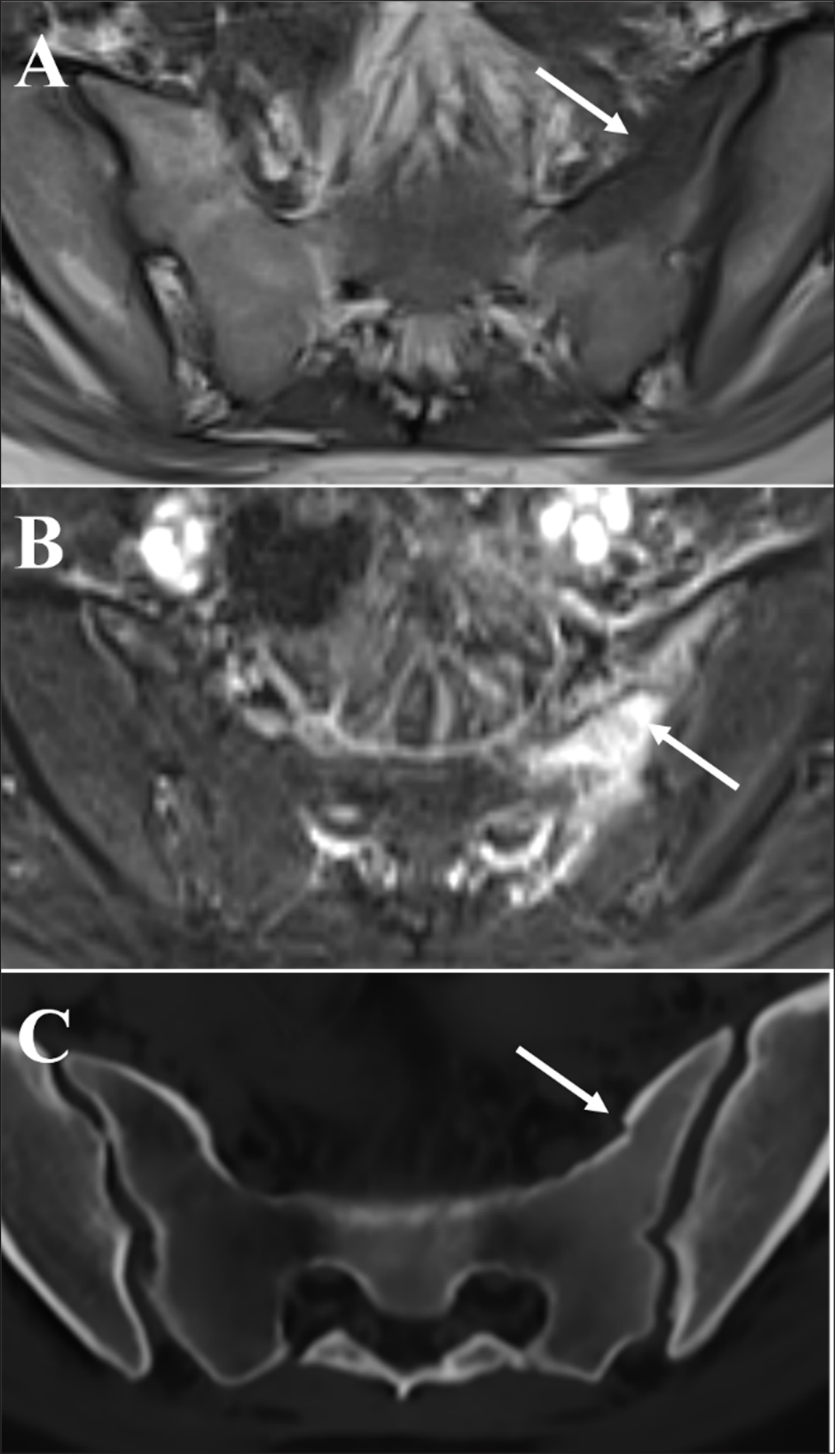


Since she suffered from neurological complaints, a pelvic MRI scan was performed. Sacral bone marrow edema was seen on T1- and fat-suppressed T2-weighted MR images (Figure 1A and 1B). An additional 3D sequence for synthetic CT (sCT) reconstruction was also obtained, that is, 3D T1-weighted radio-frequency spoiled multiple gradient-echo sequence (T1MGE). The reconstructed sCT images (Figure 1C) clearly depicted the sacral fracture (arrows) that was suspected due to bone marrow edema.

The sCT images were reconstructed from the 3D T1MGE images with commercially available software (BoneMRI Pelvic Region, version 1.3; MRIguidance), using a deep learning method based on CNN U-net architecture. This method exploits local spatial contextual information in the multiecho data to reconstruct the latent bone structures, which was learned using paired MRI and CT data. The resulting sCT image expresses radiodensity contrast in Hounsfield units.

## Comment

The clear depiction of the fractures on the sCT images is largely due to the detailed delineation of the cortical bone. The excellent cortical delineation of synthetic CT has been first demonstrated in the imaging of the sacroiliac joints: sCT had higher diagnostic performance in the detection of bony lesions (such as erosions) compared with routine T1-weighted MRI [1].

Currently, data for fracture detection using sCT imaging is based solely on observational case studies and validation for use of the technique in trauma patients is still mandatory. Nevertheless, this case demonstrates the potential of sCT imaging as a harmless, radiation-free alternative to classic CT imaging to (re)evaluate fractures or other lesions of the cortical bone. The absence of ionizing radiation, its high similarity of the sCT images with classic CT and the one-stop-shop visualization of soft tissues and bones alike make sCT imaging very appealing as a potential future technique for clinical practice.
